# Displacement of occluder after left atrial appendage closure: A case report

**DOI:** 10.1002/ccr3.8915

**Published:** 2024-05-20

**Authors:** Yan Lin, Xiaobo Mao

**Affiliations:** ^1^ Department of Cardiology Dongxihu District People's Hospital Wuhan Hubei China; ^2^ Department of Cardiology Huazhong University of Science and Technology Tongji Medical College Affiliated Union Hospital Wuhan Hubei China

**Keywords:** atrial fibrillation, displacement of occluder, left atrial appendage occluder, left atrial appendage occlusion (LAAC)

## Abstract

**Key Clinical Message:**

Atrial fibrillation is closely associated with thrombotic events. In non‐valvular atrial fibrillation, 90% of thrombi are formed by the left atrial appendage. Left atrial appendage occlusion (LAAC) can effectively prevent the detachment of left atrial appendage thrombus during atrial fibrillation, thereby reducing the risk of long‐term disability or death caused by thromboembolic events. However, the identification and management of complications in LAAC are also very important.

**Abstract:**

The efficacy and safety of left atrial appendage occlusion (LAAC) in preventing non‐valvular atrial fibrillation stroke have been confirmed by multiple randomized controlled and registered studies, and have been recommended by several guidelines for stroke prevention in patients with atrial fibrillation at high‐risk of stroke. We reported an 80‐year‐old male patient with persistent atrial fibrillation. The patient underwent left atrial appendage closure surgery due to high risk of embolism and bleeding. On the second day after surgery, echocardiography showed displacement of the left atrial appendage occluder. Immediately perform removal of left atrial appendage occlude and left atrial appendage occlusion on the same day, and the patient was discharged on the fifth day after surgery without any special circumstances. This case demonstrates the feasibility and important clinical significance of using interventional surgery to remove the left atrial appendage occluder after displacement in clinical practice.

## 1 INTRODUCTION

Atrial fibrillation (AF) is a common atrial arrhythmia, associated with increased incidence rate and mortality of cardiovascular events.^1^ AF is associated with an increased risk of thromboembolism and stroke, which is needed to long‐term anticoagulant therapy to reduce the risk of stroke. Despite progress and development in anticoagulation strategies, the high risk of bleeding complications and strict medication adherence make treatment more complex. In the past few decades, the left atrial appendage has become a promising therapeutic target, which can prevent thromboembolic events while reducing the problem of bleeding complications.^2^, ^3^ The 2023 SCAI/HRS guidelines suggest that LAAC is suitable for non‐valvular atrial fibrillation, and the CHA2DS2 VASc score is ≥2 (males) or ≥3 (females). In addition, patients should have a high risk of bleeding (e.g., high HAS‐BLED score ≥3) or oral anticoagulant(OAC) intolerance(including previous bleeding, risk of falls, uncontrolled hypertension, kidney or liver failure, alcohol intake, concurrent use of antiplatelet or non steroidal drugs, high‐risk occupations, poor compliance, unstable international standardized ratios, OAC allergies, and drug interactions, among other factors).^4^ Research has shown that LAAC surgery is associated with a reduced incidence of cardiovascular events during hospitalization, and can also improve patient prognosis in long‐term follow‐up.^5^, ^6^ In the past 20 years, among non‐valvular atrial fibrillation patients with increased risk of stroke, LAAC has become a safe and effective alternative to oral anticoagulants for stroke prevention.^7^ However, the drawbacks of left atrial appendage occlusion cannot be ignored. First, the complications of surgery include occluder thrombosis, pericardial embolism, perioperative embolism, and occluder detachment. In addition, the impact of left atrial appendage occlusion on the heart should also be considered. The left atrial appendage has important physiological functions, including mechanical and endocrine functions, and also participates in electrophysiological activities.^8^, ^9^, ^10^

## 2 CASE HISTORY/EXAMINATION

An 80‐year‐old male patient was admitted 2 days after sudden syncope. Admission blood pressure: 98/67 mmHg, heart rate 86 beats/minute. The duration of atrial fibrillation is unknown (CHA2DS2‐VASc:6 points, HAS‐BLED: 4 points), and long‐term treatment with rivaroxaban anticoagulant therapy. Previous history of gastric bleeding, Have a long history of drinking alcohol. The patient refuses to continue long‐term oral anticoagulants. After Admission, esophageal echocardiography ruled out intraluminal thrombus. Dynamic electrocardiogram: The average heart rate is 66 bpm, the slowest heart rate is 30 bpm; the fastest heart rate is 139 bpm, A total of 92,033 cardiac beats were analyzed; long RR intervals greater than 2000 ms were 67, and the longest is 2562 ms. Total atrial fibrillation. Cardiac ultrasound showed enlargement of the left atrium (4.4 cm), enlargement of the right atrium (5.4 cm), and enlargement of the right ventricle (4.3 cm), left ventricular ejection fraction: 58%. Head Magnetic resonance imaging shows no obvious abnormalities. Diagnosis: (1).Persistent atrial fibrillation, (2). Transient ischemic attack, (3).coronary atherosclerosis, myocardial bridging, (4). Grade 1 hypertension (high‐risk group).

## 3 METHODS

### 3.1 Differential diagnosis

The differential diagnosis of syncope in patients includes cardiogenic syncope and vasovagal syncope.

Transient ischemic attack can also manifest as transient syncope with a short duration, and no abnormal signs can be observed on head CT.

### 3.2 Treatment

**FIGURE 1:**
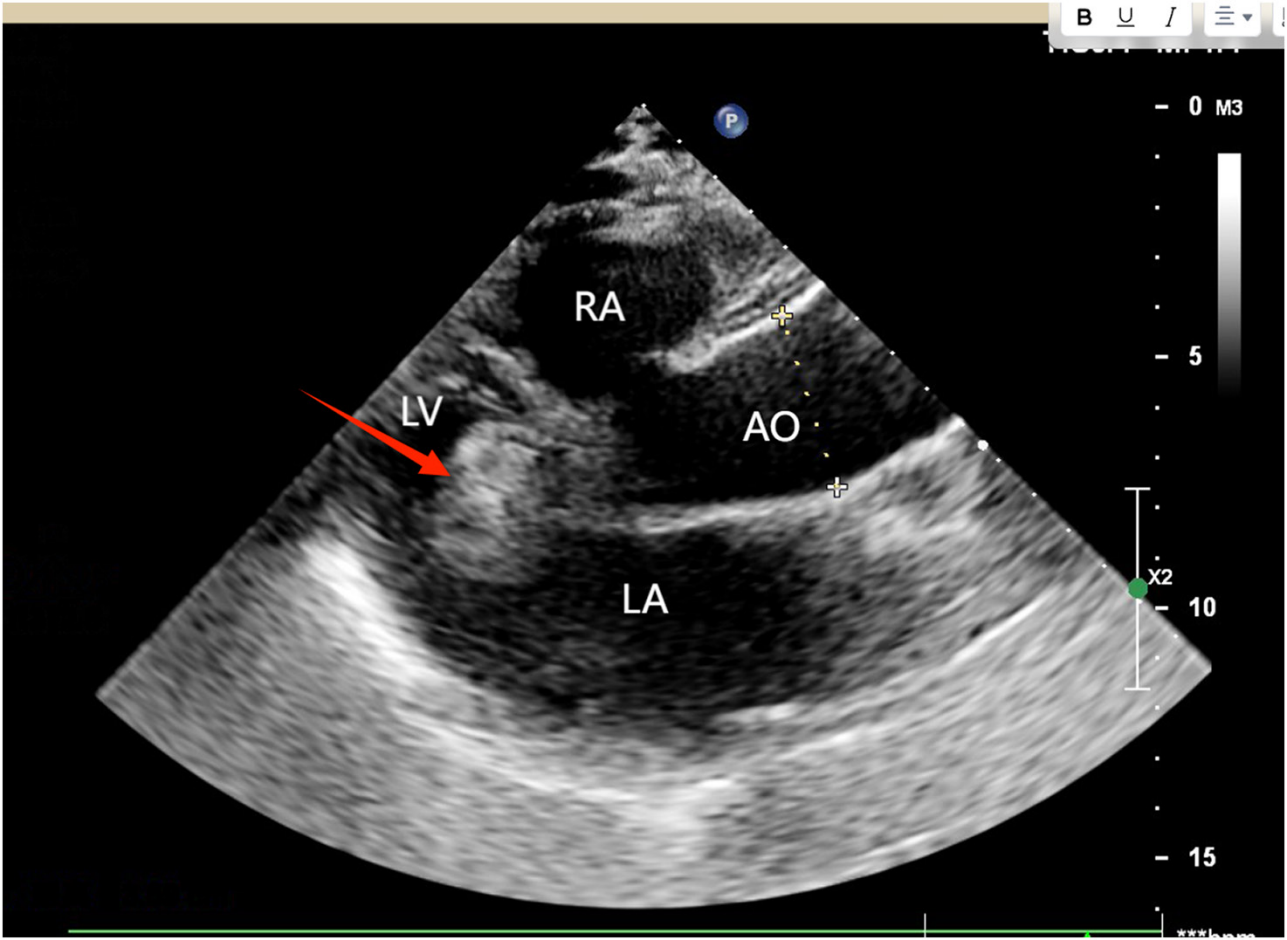
Echocardiography indicates occlusion device displacement (The occluder is located in the left ventricle).

**FIGURE 2:**
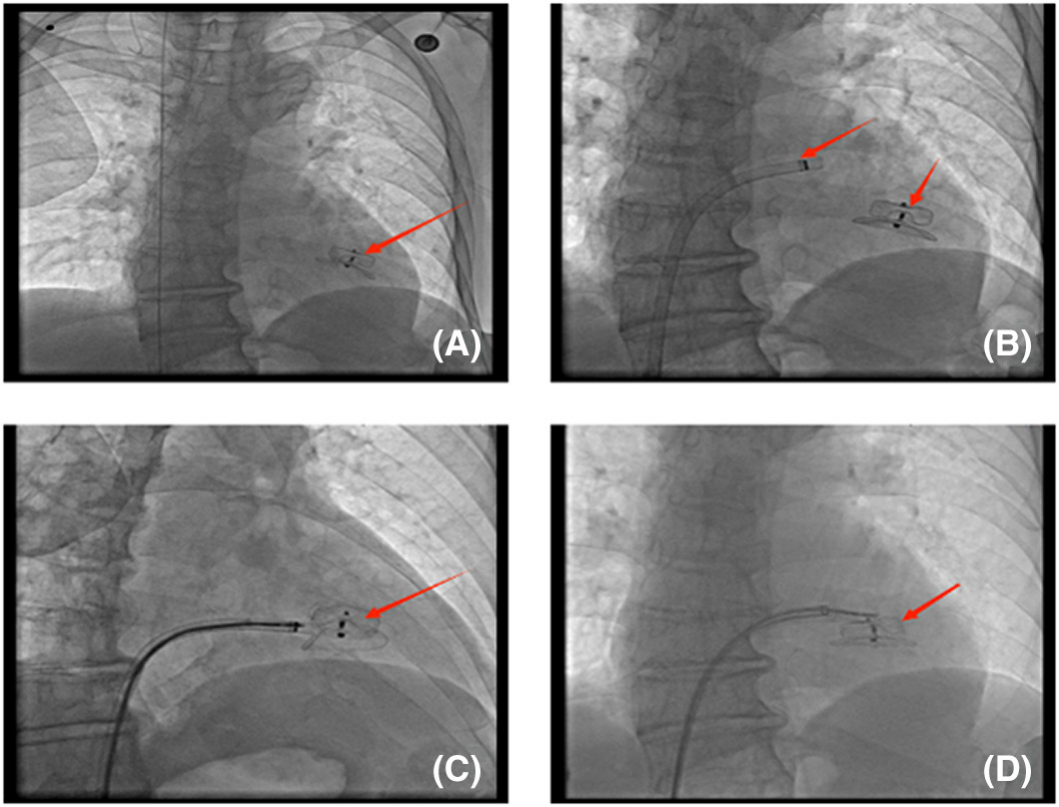
Adjustable bending sheath insert the left ventricle and perform occluder grasping. (A) Left ventricular septal occluder detachment; (B) Medtronic 4FC12 adjustable bending and Displaced occluder; (C, D) Grasp the occluder into the adjustable bending sheath and successfully remove the occlude.

After admission, the patient underwent LAAC intervention surgery and was equipped with an LACbes 26 mm * 32 mm left atrial appendage occluder according to the size of the patient's left atrial appendage, and there were no significant abnormalities after the surgery. On the first day after surgery, echocardiography revealed abnormal echogenicity in the left ventricular cavity, which is considered occluder displacement(Figure 1). Consider displacement caused by a small occluder size. Puncture through the right femoral vein, insert a blood vessel sheath, send a long sheath tube, atrial septal puncture needle to the superior vena cava, and retract to the atrial septum, after successful RAO45° atrial septal puncture, Medtronic 4FC12 adjustable bending sheath was inserted, insert the 7F AL1 catheter along the flexible sheath to the left ventricle and perform occluder grasping (Figure 2), after successfully grasping the occluder, push and inject ice salt water along the sheath to soften the occluder. After fully softening the occluder, smoothly grasp the occluder into the adjustable bending sheath and successfully remove the occlude. The patient's vital signs are stable and there is no special discomfort, continue with LAAC surgery. Send the pigtail catheter along the outer sheath to left atrial appendage angiography for examination, and measure the inner diameter and opening diameter of the left atrial appendage, According to the size of the patient's left atrial appendage, LACbes26mm * 32 mm left atrial appendage occlusion umbrella is configured in vitro. Release of occlusive umbrella under RAO30 + CAU20 imaging, Under X‐ray, the occlusive umbrella is stable at the opening of the left atrial appendage, and angiography shows isolation of blood flow between the left atrial appendage and the left atrium. Perform another traction test for 1 min, RAO30 + CAU20 confirmed that the sealing umbrella was firmly fixed, and there was no obvious leakage of contrast agent around the umbrella, indicating satisfactory sealing, echocardiography examination showed that the occlusive umbrella was stably fixed at the opening of the left atrial appendage, and there was no signal from the left atrial appendage or left atrial septum around the umbrella, indicating a successful closure of the left atrial appendage.

## 4 CONCLUSION AND RESULTS

The patient was discharged on the fifth day after surgery. Regularly taking rivaroxaban anticoagulant treatment after discharge, and switching to dual antiplatelet therapy 3 months later. Replace with single antiplatelet therapy after 6 months of dual antibody treatment. The patient came to the outpatient clinic for follow‐up 1 month after surgery. Echocardiography indicates good placement of the occluder and no abnormal thrombus formation in the heart cavity.

## 5 DISCUSSION

The detachment of the occluder is one of the most serious complications of LAAC surgery, often occurring during the perioperative period. The reason for the dislocation of the occluder in this patient is considered to be that the occluder umbrella is too small, resulting in an unstable attachment, The second surgery was successful after replacing the left atrial appendage occlusion umbrella with LACbes26mm * 32 mm, and no obvious abnormalities were found in the postoperative follow‐up echocardiography. The main reasons for the detachment of the occlude include: (1) The size of the occluder is too small relative to the diameter of the auricle, (2) The occluder is placed too far out and not firmly fixed, (3) The pre‐installation of the occluder is not firm, or the screw at the connection between the push rod and the occluder occurs after the occluder is fully recovered. When the intervention method for removing the occluder is expected to be difficult or carries significant risks, it is recommended to perform cardiac surgery for removal. This article provides new treatment experience for clinical practice by using an intervention plan to remove the detached occlude. In early LAAC surgical experience; the incidence of complications such as pericardial tamponade, occluder displacement, and thromboembolism is relatively high. In the past decade, improved operational experience, meticulous techniques, and improved equipment iteration have greatly reduced the incidence of major complications.^7^ Although the current indications of LAAC mainly focus on non‐valvular atrial fibrillation patients and contraindications for oral anticoagulation, it is almost certain that the future will expand the indications, applicability, and scope of use of these devices.^11^ Moreover, different designs of the left atrial appendage occluder may affect prognosis (such as detachment or embolism). The advantage of devices without an external disc is that it has less interference with surrounding structures and a smaller surface that interacts with blood.^8^ At present, research has discussed the use of virtual reality (VR) derived from cardiac computed angiography data to predict the size of LAAC devices. VR visualization of the left atrial appendage opening from different perspectives can better understand its funnel‐shaped structure. The VR measurement of the maximum opening diameter has the strongest correlation with the diameter of the insertion device. Therefore, VR may provide new imaging possibilities for evaluating complex preoperative structures such as the left atrial appendage. To prepare suitable occluder before LAA surgery and reduce the occurrence of occluder detachment.^12^

## AUTHOR CONTRIBUTIONS

**Xiaobo Mao:** Writing – review and editing. **Yan Lin:** Writing – original draft.

## FUNDING INFORMATION

None declared.

## CONFLICT OF INTEREST STATEMENT

There is no conflict to be declared.

## CONSENT

The authors confirm that written consent for submission and publication of this case report including images and associated text has been obtained from the patient.

## Data Availability

Data sharing is not applicable to this article as no new data were created or analyzed in this study.
